# Modeling unit non-response and validity of online teaching evaluation in higher education using generalizability theory approach

**DOI:** 10.3389/fpsyg.2023.1202896

**Published:** 2023-09-04

**Authors:** Yayra Dzakadzie, Frank Quansah

**Affiliations:** Department of Educational Foundations, University of Education, Winneba, Ghana

**Keywords:** evaluation, teaching quality, non-response, validity, generalizability theory (GT), reliability

## Abstract

**Introduction:**

Unit non-response is a common phenomenon in online teaching evaluation in higher education institutions. However, little is known about the relationship between the rates of unit non-response and the quality of online teaching evaluation exercise. This study explored the incidence of unit non-response and how this phenomenon relates to the reliability of students’ responses to online teaching evaluation.

**Methods:**

Adopting the generalizability theory approach, students’ evaluation of teaching data from a university in Ghana was analyzed by conducting both generalizability study (G-study)- and decision study (D-study) analyses.

**Results:**

The results revealed that unit non-response among students was predominant in online teaching evaluation exercise. The study demonstrated that higher rates of non-response among students were associated with high levels of measurement errors and low reliability of responses.

**Discussion:**

The findings of this study have implications for the accuracy of online evaluation data obtained for decision-making in higher education contexts. The study calls on higher education administrators to embark on sensitization and awareness campaigns that target students on the need to actively participate in the appraisal of teaching at the university to address the issue of unit non-response.

## Introduction

Student evaluation of courses and teaching is a common phenomenon in higher education institutions that has been in existence for ages (Spooren and Van Loon, 2012). In recent times, however, several higher education administrators have switched from the traditional mode of administration (paper-and-pencil) to the online mode to reduce costs, improve data-gathering practices and make data analysis easier (Groves et al., 2009). This transition has also led to decreased rates of student participation, resulting in high rates of non-response (Dillman et al., 2002). Several studies have shown that unit non-response (i.e., individuals not responding to any item on the survey instrument or complete non-participation) in online evaluation surveys is a key challenge for the introduction of technology in students’ appraisal of teaching and learning (Guder and Malliaris, 2013; Marcham et al., 2020; Čehovin et al., 2022; Falk and Thies, 2022; Plante et al., 2022).

Non-response in students’ evaluation of teaching via the online mode has been increasing in recent times (Adams and Umbach, 2012); thus, the smallest number of non-response should be studied for several reasons. Primarily, a unit of non-response results in a response bias that affects the quality of the data obtained. Non-response bias is an error that occurs when there is a systematic change between persons who respond to the survey instrument and those who do not (McDaniel and Gates, 2012). In several instances, non-response in a survey is non-random (Dillman et al., 2009), suggesting that the non-response occurred for a reason. As students’ non-response during an evaluation exercise increases, the probability that non-participants’ views will vary from actual participants’ judgments also increases (Adams and Umbach, 2012). Thus, the accuracy (validity) of teaching evaluation data may be questioned if participants who failed to complete the evaluation survey systematically differ from those who completed the survey (McDaniel and Gates, 2012; Reisenwitz, 2016). The effects of unit non-response cannot be less emphasized because results from the students’ appraisal of teaching survey are generalized to the population and, consequently, a reflection of the views of all eligible participants in the survey (Groves et al., 2009).

It appears that the utilization of data from students’ evaluations of teaching can be flawed by the unit non-response phenomenon. With a declining response rate, the worth of data and its use happens to be in jeopardy (Groves et al., 2009). Thus, the quality of such data matters in higher education institutions, particularly because they have numerous uses including promotion, reappointment, and instructional management decisions (Porter and Whitcomb, 2005). In this era of decision-making based on data, it is vital to gather data that reflect the views of the larger population for sound decisions to be made. Issues on student participation rate in assessing the quality of teaching evaluation can be used to promote institutional changes and redefine new strategies in institutions of higher education.

### Selection bias model

According to the selection bias model (Gronau, 1974; Heckman, 1974), selection bias occurs when observations of interest are tied to a non-randomly selected subpopulation. In most cases, the characteristics of this subpopulation may go unnoticed or may be noticed after the outcome of interest is observed. This situation translates into the problem of data missing-not-at-random (Rubin and Little, 1987; Allison, 2001; Berg, 2005). The selection bias model provides insight into the variabilities surrounding the presence of high unit non-response in teaching evaluation exercise. The model projects that the prevalence of high unit non-response suggests that the evaluation results represent the views of non-random students who participated in the survey based on some extraneous factors such as evaluators’ perception of anonymity of the data and motivation. The decision on whether students will participate in the evaluation survey or not is based on their net utility derived from the response. That is, the satisfaction students derive from responding to the course and instructor evaluation survey reinforces their decision to participate. Students who do not derive satisfaction from responding to the evaluation are unlikely to participate in the survey and vice versa. The difficulty in ascertaining an estimate of this net utility impedes the knowledge about which category of students are unwilling to participate in the evaluation of teaching (Heckman, 1974).

The prevailing literature on students’ evaluation of teaching has revealed that students who are high achievers (using cumulative grade point averages or grades) and, in most settings, female students have a greater probability of participating in evaluation surveys (Porter and Whitcomb, 2005; Avery et al., 2006; Porter and Umbach, 2006; Marsh, 2007; Kherfi, 2011; Spooren and Van Loon, 2012). In addition, the majority of evaluation surveys are carried out close to the end of the semester, and thus, students might have already received their scores/grades for some classroom assessment; this might influence their decisions to respond to the evaluation survey. Moreover, survey fatigue can decrease participation rates, where the cause of fatigue is the result of responding to many surveys surrounding similar issues simultaneously and/or lengthy evaluation items (Groves et al., 2004; Spooren and Van Loon, 2012). Likewise, in Ghanaian universities, the evaluation of teaching is conducted around the same time (i.e., getting to the end of the semester). Student evaluators might end up not responding at all or, in the worst case, provide inaccurate ratings due to fatigue. Consequently, evaluation scores may be misrepresentative of the students’ opinions regarding the evaluation objects (i.e., lecturers) because students who participated in the survey might possess some characteristics different from those who failed to participate. Suppose that the majority of high-achieving students respond to the evaluation of their instructor; there is a potential selection bias even though the investigator might not be in the known. In such a situation, the evaluation data will reflect the opinions of high-achieving students rather than the views of the entire class.

### Theoretical framework: generalizability theory

Generalizability Theory (GT) is an arithmetic theory concerned with the reliability of behavioral measurements. GT is an extension of Classical Measurement Theory (CMT) (Cronbach et al., 1972). The CMT operates on the assumption that each test score or observation comprises a true score and an error score that generates a single dependability coefficient for a set of equivalent observations. On the one hand, this hypothesis may be practical when the parallel forms are equalized cautiously; on the other hand, it becomes unrealistic where variances or average scores are dissimilar or when there are heterogeneous items on the test form. From the perspective of internal consistency, reliability appears to be low in a multidimensional measurement; nevertheless, parallel forms and test–retest reliability estimates may be simultaneously high at the same time. Cases such as the aforementioned contradictions and restrictions of the CMT model of reliability facilitated the introduction of the GT which uses a less rigid approach that eliminates these restrictions and helps in the analysis of errors emanating from potential sources of variability such as tasks, raters, items, and time. The GT framework combines the different sources of variation and simultaneously computes an all-inclusive dependability/reliability estimate. Furthermore, GT removes the traditional variations between validity and reliability (Allal and Cardinet, 1997). Due to the advantages of the GT over the CMT, it has been applied in several studies to address research problems in different areas of study (García-García et al., 2013; Morales-Sánchez et al., 2020; Reigal et al., 2020; Cobbinah et al., 2022).

Shavelson and Webb (1991) argued that GT is a protracted form of the CMT for four reasons: 1) The GT has the ability to estimate several sources of variability in a single computation, 2) the use of GT guarantees the estimation of the magnitude of each source of variability, 3) GT permits the computation of two different errors of measurement and reliability coefficients and thus, makes it possible to take relative and absolute decisions, and 4) GT allows for realistic measurement decisions to be made to reduce measurement errors to the barest minimum based on specific purposes.

Notably, factors such as time, tasks or items, and raters are known as facets or sources of variability in the framework of GT (Brennan, 2001b). In other words, the facet is a concept that reflects all sources of probable measurement errors. Therefore, it is preferable to reduce the degree of variation related to the source of variation as much as possible (Alharby, 2006). Every source of variability has levels that are referred to as measurement conditions. For example, for this study, 10 items were used to measure the teaching construct, and thus, the item facet had 10 measurement conditions. Similarly, the rater facet had over 2,700 raters and thus, the conditions of measurement for the rater facet were over 2,700. In general, the potential conditions of measurement for any random facet are deemed infinite in magnitude. The selected conditions of measurement that are admissible to the investigator are known as the universe of admissible observations.

**Figure 1:**
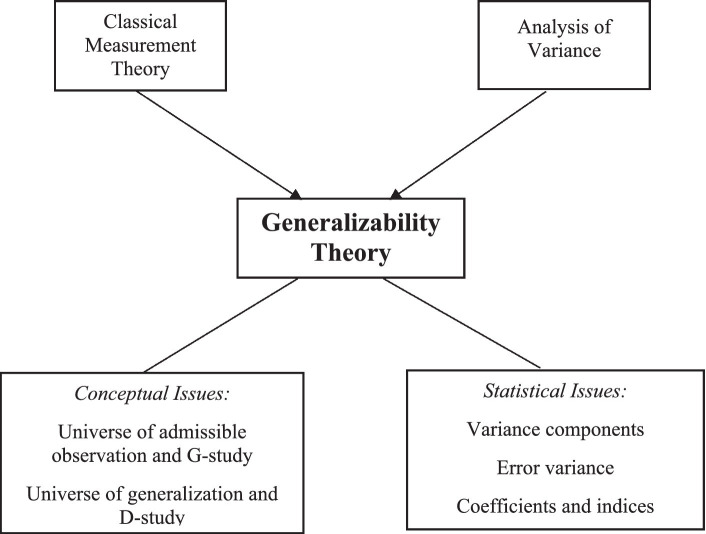
Parents and conceptual framework of GT. A chart showing the various components of the GT, including the conceptual and statistical dimensions.

Another concept worthy of explanation is the universe of generalization. This concept signifies the set of conditions to which an investigator wishes to generalize. In simple terms, people act as the focus of the measurement based on which conclusions are made. Consequently, persons are not considered as a source of variability because variations contingent on persons are always preferred. The universe score is another concept that needs to be explained. By definition, a universe score is a measurement score that reflects the average of the scores attained from the universe of admissible observations for the sources of variation. The universe score variance is analogous to the true score variance as used in the framework of CMT; hitherto as dissimilar from it, two distinct error variances are obtained in the GT framework. This difference originates from the notion that GT allows for two decisions to be made. Both absolute and relative error variances are computed and interpreted in the GT context. In this case, the relative error variance is analogous to the error variance in the CMT framework (Shavelson and Webb, 1991). Even though CMT and analysis of variance (ANOVA) are seen as the parents of GT, the child is equally more and less than the simple combination of its parents, and understanding GT necessitates insight into more than its ancestry (Figure 1).

There are two phases in the GT framework: a generalizability study (G-study) and a decision study (D-study). A G-study aims to compute variance component estimates associated with a universe of admissible observations (Brennan, 2001a). The D-study, on the other hand, focuses on finding strategies for reducing measurement errors by using information obtained from the G-study (Crocker and Algina, 1986; Shavelson and Webb, 1991; Brennan, 2001a). The D-study emphasizes the computation, utilization, and interpretation of variance components for making a decision with well-designed measurement approaches. Conceivably, the most significant D-study consideration is the description of a universe of generalization, which is described as a universe to which the investigator wishes to generalize depending on the outcome of the specific measurement process (Shavelson and Webb, 1991).

The use of the GT approach in this study is justified in its approach to modeling the relationship between unit non-response and the reliability of online teaching evaluation outcomes. The GT approach has an advantage over other procedures (like CMT and many-facet Rasch model) in terms of providing useful group-level information (i.e., internal consistency of the instrument and inter-rater agreement in rating teaching), especially when teaching evaluation data are analyzed based on classes in relation to the instructor. More importantly, the GT estimation procedure has the ability to estimate the degree of measurement error and reliability (both relative and absolute) associated with varied levels of the conditions of measurements of the facets under investigation. In other words, this approach provides information on the extent of measurement errors (or reliability) in the ratings when the number of students reduces (low participation) or increases (high participation) (Alharby, 2006).

### The present study

The higher education system, together with the teaching, learning, and assessment practices in Ghana, are similar to those of other countries worldwide (Quainoo et al., 2020). Lecturers in various institutions are assigned courses to engage students for 10–15 weeks. Depending on the institutional policy and the type of program of study, students are taken through a series of in-class and out-of-class training accompanied by diverse forms of assessments used at different times within the teaching and learning duration (Quansah et al., 2019; Nugba and Quansah, 2020; Quansah and Ankoma-Sey, 2020). At the end of the semester, examinations are usually organized for students who registered for the courses, although other formative assessments are conducted. Before the examination, the students are allowed to evaluate their experiences with teaching and learning. The outcome of this evaluation exercise, normally conducted through online means (e.g., student portals, Moodle platforms), is used by university administrators to make decisions concerning program modification, promotion and tenure, and professional development training. Meanwhile, the outcome of the evaluation is made available to the respective lecturers for discussion at the departmental/faculty level.

Due to the high-stakes nature of appraisal data, biases that threaten the validity of the information provided have been studied extensively across geographical boundaries (Spooren et al., 2013; MacNell et al., 2015; Hornstein, 2017; Quansah, 2020; Kreitzer and Sweet-Cushman, 2021; Quansah, 2022; Stoesz et al., 2022). These previous studies have stressed that the sources of variation in students’ appraisal of teaching are attributed to raters (i.e., students), items, occasions, course types, and teacher characteristics unrelated to teaching. Meanwhile, the increasing rate of non-response to online teaching evaluation survey in higher education have been argued to also influence the validity of teaching evaluations (Adams and Umbach, 2012; Guder and Malliaris, 2013; Marcham et al., 2020; Čehovin et al., 2022; Falk and Thies, 2022; Plante et al., 2022).

Given the consequences of non-response, some scholars have attempted to investigate the nexus between unit non-response and variabilities in student responses (Bacon et al., 2016; Reisenwitz, 2016; Goos and Salomons, 2017; Luo, 2020). These earlier studies adopted two approaches to their investigation where: (1) responses were compared for students who identified themselves as non-respondents to previous evaluation surveys and those who reported being regular participants and (2) responses of classes with high unit response rates and those with low unit response rates were compared. Although these studies have discovered significant disparities in the evaluation results between the identified parties in each study, it is unclear which of the two parties provided accurate responses. Additionally, concerns about the estimated levels of validity and reliability which the (high/low) response rates contribute to the measurement of teaching quality are not well understood. In this study, we sought to model response rates to the measurement errors and reliability of responses during a teaching evaluation survey. In this vein, two objectives guided the research: (1) to explore the prevalence of students’ unit non-response rates in evaluating teaching and learning and (2) to examine how unit non-response influences the reliability of data on students’ evaluation of teaching.

## Materials and methods

### Study design

The basic design used in this study was a two-facet partially nested random design. A facet is a set of related measurement conditions (Brennan, 2001b). For example, an item was considered a facet in this study. Similarly, the rater (i.e., student) also served as a facet. Although the object of measurement (i.e., lecturer) had several measurement conditions, it was not considered a facet, as indicated in the GT framework (Shavelson and Webb, 1991). This explains why the two-facet design was adopted. The sources of variations are labeled as follows: lecturer (i.e., the object of measurement) was symbolized as ***p***; student (i.e., rater) was symbolized as ***r***; and the item was symbolized as ***i***.

Generalizability Theory (GT) designs can be crossed (*x*), nested (:), or a combination of both. A design is crossed when all the conditions of measurement in a particular facet, say item facet, are observed with all the conditions of measurement of another source of variability (e.g., raters) (Shavelson and Webb, 1991). For example, students (i.e., raters) may be required to rate their classroom teachers (i.e., persons/lecturers) on the quality of their instruction. If the investigator is interested in a single facet, such as the rater, then the design will be “persons crossed with raters” (*p x r*). This means that all raters assessed the teachers’ quality of teaching. However, if there is an additional facet (such as item), then the design will be persons crossed “with raters crossed with items” (*p* × *r* × *i*). Designs considered nested are adopted when two or more conditions of measurement of one facets are observed with the condition of measurement of another facet(s) (Shavelson and Webb, 1991). For instance, in this study, the rater facet was nested in the object of measurement (i.e., lecturers) because different students rated different lecturers based on their teaching.

Furthermore, the GT design can be fully or partially nested (Brennan, 2001b). This study employed a partially nested design because not all facets were nested in the object of measurement (i.e., the lecturer). That is, each lecturer was rated using the same items, although different students rated different lecturers. Likewise, all facets in this study were considered random because the sample size (conditions of measurement) was much smaller than the magnitude of the universe, and the sample was either randomly drawn or deemed as replaceable with any other sample of the same size selected from the universe (Brennan, 2001b). For instance, the item facet is considered random when the items used are not exhaustive and other items can be added to perform the same function. That is, the items used in that particular study are just a sample of all items that can function in a similar way. In other words, if in a GT study, there are 13 raters and there are other raters who can perform the same role and can be employed to either replace or add to the existing raters, then the rater facet is random.

For a GT design to be considered balanced or unbalanced lies in whether the design has no missing information, and for any nested facet, the size of the sample is unequal or constant for each level of that facet (Brennan, 2001b). In particular, this study adopted an unbalanced design because the nested facet was unequal across the object of measurement. That is, the students who rated the lecturers differed from one lecturer to another. In this regard, the students were nested within lecturers.

Consequently, the aforementioned GT design is symbolized as (***r**:**p***) *x* ***i***. Based on the two-facet partially nested random design, the observed score for one instructor can be decomposed as follows:


$$X_{pir}=\mu$$

$$+\left(\mu_{p}-\mu \right)$$

$$+\left(\mu_{i}-\mu \right)$$

$$+\left(\mu_{pr}-\mu_{p}\right)$$

$$+\left(\mu_{pi}-\mu_{p}-\mu_{i}+\mu \right)$$

$$+\left(X_{pri}-\mu_{pi}-\mu_{pr}+\mu_{p}\right)$$

$$\sigma^{2}\left(X_{pri}\right)=\sigma_{p}^{2}+\sigma_{i}^{2}+\sigma_{r.pr}^{2}+\sigma_{pi}^{2}+\sigma_{ri.pri,e}^{2}$$

The two-facet partially nested random design [(***r*: *p***) *x* ***i***] has five sources of variability: person (*p*), item (*i*), items crossed with persons (*pi*), raters nested in persons (*r*:*p*), and raters nested in persons crossed with items (*r:p*) *i,e.*

### Participants

This study used secondary data on online teaching evaluation obtained from a university in Ghana. The evaluation data comprised teaching appraisal ratings provided by students enrolled on various programs within the university. All cases and data points were included in this study. Thus, the sample size was 24,726 regular students, corresponding to 152,658 expected responses (based on the number of courses taken) from 1,673 courses. It should be noted that students took different courses and consequently, rated lecturers teaching the different courses. Of the 152,658 expected responses, only 73,906 were received from the students. That is, not all the students responded to the online evaluation form. The available cases were included in the study using a census approach. Although the data obtained had over 20 items, only 10 items were extracted for this study because they focused on soliciting information regarding the quality of teaching and teaching strategy. Students responded to the evaluation form in a manner that required answers for each section; otherwise, they could not open the subsequent sections to answer. This suggests that there were no item non-responses in the dataset.

The data obtained for this research were screened and cleaned based on four criteria: (1) courses that had only one student response were deleted from the final data set because there would be no variance; (2) core courses were deleted from the analysis. The reason is that although several instructors taught the general/core course, the ratings were merged to appear as if only one instructor handled the course; (3) duplicated courses were deleted because this could confound the findings of the study; and (4) specific courses that had inconsistencies in terms of the responses/data were deleted. After the implementation of these criteria, 2,553 students (within 145 courses) remained in the dataset for final analysis through purposive sampling.

### Instrument

The study relied on secondary information from the online teaching evaluation conducted by the selected university. This suggests that this research did not directly make use of any instrument. However, the secondary data retrieved were information based on an evaluation instrument, which was designed and administered by the university in question. This evaluation questionnaire comprised 25 items sectioned into five domains (i.e., course outline, facilitators’ class attendance, mode of delivery, assessments conducted and strengths/weaknesses). In the context of this study, data on the mode of delivery section was only accessed and used because that domain contains items and response options which have high levels of subjectivity (e.g., *“The lecturer demonstrated knowledge of the subject matter*,” “*The lecturer’s delivery was well organized and systematic*”). The “mode of delivery” section has 10- items with response options “*Not very well*,” “*Not well*,” “*Well*” and “*Very well*.” The other sections have items that required objective responses (e.g., “*The lecturer made a course outline available to students at the beginning of the course*,” “*The number of assessments given by the lecturer was*….”). The last section (i.e., strengths/weaknesses) required students to write the lecturer’s strengths and weaknesses which did not qualify for this kind of study due to the qualitative nature of the data.

The evaluation form has an instructional text that informed the respondent that every student is required to complete the survey for all their registered courses. Despite this information, the online teaching evaluation exercise in the selected university is not compulsory; however, the online system does not permit an incomplete form to be submitted. This means that it is either the student submits a completed evaluation form or does not entirely participate in the exercise. The instructional text also indicates that the names and index numbers of respondents are kept anonymous and that participating in the survey would help the university with valuable feedback for improving teaching and learning activities.

### Procedure

Ethical clearance was obtained from the Institutional Review Board (IRB) of the University of Cape Coast (UCC). The IRB of the UCC is an independent and credible body that reviews proposed research, adhering to all ethical standards of the sixth revision of the Helsinki Declaration. A letter was drafted by the corresponding author to officially seek permission to access and use data. Copies of these letters were delivered to the officer in-charge of the evaluation exercise to formally seek permission to access and use data. This event followed an initial contact with the head of the unit and other staff to discuss what the study sought to achieve and the need for the study (Creswell and Guetterman, 2019).

## Statistical analyses

Analyses were conducted in the R-studio environment using the *gtheory* package (see Huebner and Lucht (2019), https://cran.r-project.org/package=gtheory). The first objective was addressed by computing the frequency counts and percentages. Two phases of analyses were performed to address the second research objective: G- and D-study analyses. At the first stage of the G-study analysis, ANOVA analysis was performed using the “*aov*” function in R-studio in order to estimate the degrees of freedom, sum of squares and mean squares for the data. The “*gstudy*” function was then utilized to obtain the estimates for the two-facet partially nested random design (i.e., variance components related to the universe of admissive observation for the respective facets) (see Table 1). It must be highlighted that, prior to the analysis, the data for the analysis was transformed from the “wide” form to the “long” format to make the data compatible with the analysis to be performed.

**Table 1:** Sources of variance and their expected mean square formula for two-facet, partially nested random unbalanced G-study.

Sources of variation	Variance component	Expected mean square
Person (*p*)	$\sigma_{p}^{2}$	$n_{r}n_{i}\sigma_{p}^{2}+n_{i}\sigma_{r.pr}^{2}+\sigma_{ri.pri,e}^{2}$
Item (***i***)	$\sigma_{i}^{2}$	$n_{p}n_{r}\sigma_{i}^{2}+n_{r}\sigma_{pi}^{2}+\sigma_{ri.pri,e}^{2}$
Person x item (***pi***)	$\sigma_{pi}^{2}$	$n_{r}\sigma_{pi}^{2}+\sigma_{ri.pir,e}^{2}$
Rater: person (***r*:*p***)	$\sigma_{r.pr}^{2}$	$n_{i}\sigma_{r.pr}^{2}+\sigma_{ri.pri,e}^{2}$
(Rater: person) x item (***r**:**p***)***i,e***	$\sigma_{ri.pri,e}^{2}$	$\sigma_{ri.pri,e}^{2}$

In the second phase of the GT analysis, known as a decision study (D-study), information from the variance components of the G-study was used to design measurement procedures to understand the level of precision and errors when the number of raters is varied. The optimization analysis was performed using the “*dstudy*” function (Shavelson and Webb, 1991; Marcoulides, 2000; Brennan, 2001b, 2011). Since the study focused on the number of raters (i.e., students), the item facet was held constant such that only the number of students was varied. Two strategies were used to modify the number of raters: (1) an interval of 10-rater difference was used for an optimization varying the number of raters from 0 to 100 after which the measurement errors and reliability coefficients were examined, (2) the mean non-response estimate was computed across all the classes. The number of raters was then varied in the model based on this mean non-response estimate. The optimization design for the measurement design is [(***r*: *p***) *x* ***i***] with a fixed ***i*** (number of item) while modifying the number of observed levels of the rater facet.

Four key indicators were the focus of the GT analyses: absolute error variance, relative error variance, generalizability (*g*) coefficient, and dependability or phi coefficient ( Φ ). These indicators are explained as follows:

### The absolute error variance

All variance components, excluding the variance resulting from the facet of concern, known as the object of measurement and referring to lecturers in the study, add up to the error of measurement when ratings are utilized for making absolute decisions. Therefore, the absolute error variance is the summation of all variance components, except for the variance due to the object of measurement, which is omitted because it is not deemed as an error variance. Rather, it signifies systematic variance in the mean scores of the different lecturers (averaged across all raters and items) and is equivalent to the true score variance in CMT (Brennan, 2001a). For the two-facet partially nested random unbalanced design, the absolute error variance is given by the formula:


$$\sigma_{\left(\Delta \right)}^{2}=\sigma_{i}^{2}+\sigma_{pi}^{2}+\sigma_{r.pr}^{2}+\sigma_{ri.pri,e}^{2}$$

where,

$\sigma_{\left(\Delta \right)}^{2}\;$ is the symbol for absolute error variance;

$\sigma_{i}^{2}$ is the variance component resulting from items;

$\sigma_{pi}^{2}$ is the variance component resulting from persons crossed with items;

$\sigma_{r.pr}^{2}$ is variance component resulting from raters nested in person;

$\sigma_{ri.pri,e}^{2}$ is variance component resulting from raters nested in persons crossed with items plus other unknown errors.

### The relative error variance

Only those variance components that represent interactions with the object of measurement (i.e., the lecturer in this study) contribute to this error of measurement and are associated with relative decisions. The relative error variance, therefore, is the summation of all variance components within the specified model that demonstrates an interaction between the object of measurement and any facet. The square root of this estimate is equivalent to the standard error of measurement in CMT (Brennan and Johnson, 1995).


$$\sigma_{\left(\delta \right)}^{2}=\sigma_{pi}^{2}+\sigma_{r.pr}^{2}+\sigma_{ri.pri,e}^{2}$$

where,

$\sigma^{2}\left(\delta \right)$ is a symbol for relative error variance.

$\sigma_{pi}^{2}$ is the variance component resulting from persons crossed with items;

$\sigma_{r.pr}^{2}$ is variance component resulting from raters nested in persons;

$\sigma_{ri.pri,e}^{2}$ is variance component resulting from raters nested in persons crossed with items plus other unknown errors.

### The generalizability (g) coefficient

The *g*-coefficient is used for making relative decisions, and thus, is associated with the relative error variance. This is a reliability-like estimate ranging from 0 to 1.0. The higher the reliability estimate, the better the reliability of the data. It is estimated by dividing the systematic variance in the average ratings of the object of measurement (i.e., lecturer) by the sum of the relative error variance and systematic variance (Brennan, 2001a). The computation formula for estimating the *g*-coefficient is


$$g=\frac{\sigma_{p}^{2}}{\sigma_{p}^{2}+\sigma_{Rel}^{2}}$$

### The dependability or phi-coefficient

The dependability coefficient is used to make absolute decisions and thus is associated with the absolute error variance. This is a reliability-like estimate ranging from 0 to 1.0. The higher the reliability estimate, the better the reliability of the data. It is estimated by dividing the systematic variance in the average ratings of the object of measurement (i.e., lecturer) by the sum of the absolute error variance and systematic variance (Brennan, 2001a). The computation formula for estimating the phi coefficient is:


$$\Phi =\frac{\sigma_{p}^{2}}{\sigma_{p}^{2}+\sigma_{Abs}^{2}}$$

It is important to note that the two coefficients (i.e., phi and g) are exceptional cases of intra-class correlation. These two coefficients have a related structure equivalent to the structure of the reliability estimate in the CMT (Crocker and Algina, 1986). The discrepancy between the two estimates depends on the description of what constitutes an error for the decision to be made. It is essential to highlight that both the phi and *g* coefficients were evaluated using the approach of Creswell and Guetterman (2019), who indicated that researchers investigating validity issues through some correlation means should use a cut-off estimate of 0.86 and above as adequate to attain high construct validity. Based on this suggestion, we used a reliability cutoff value of 0.90.

## Results

### Prevalence of unit non-response rates in students’ evaluation of teaching

**Figure 2:**
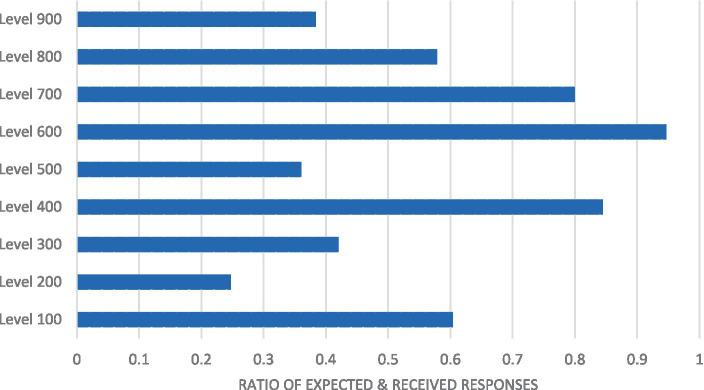
Non-response graph for levels of study. A bar graph depicting the ratio of expected and received responses against the levels of study.

The data were first analyzed to explore the unit non-response patterns by comparing the expected and actual responses, taking into consideration the levels of study. The ratio was computed for the expected and actual responses, and the ratios were compared across the levels. The ratios showed the extent of non-response such that a high across ratio estimate (closer to 1) reflected a low non-response rate, whereas a low ratio (closer to 0) depicted a high unit non-response rate. Tables 2, 3 as well as Figure 2 present details of the results.

**Table 2:** Descriptive statistics for expected and received responses.

Level of study	Expected responses	Received responses	Ratio
100	45,722	27,588	0.60
200	48,316	11,945	0.25
300	32,692	13,745	0.42
400	21,443	18,113	0.84
500	280	101	0.36
600	149	141	0.95
700	5	4	0.80
800	3,665	2,121	0.58
900	386	148	0.38
Overall	152,658	73,906	0.484

**Table 3:** Response ratio of some sampled courses.

Course (Pseudonym)	Expected responses	Received responses	Ratio	% response
ABO	3,246	6	0.001	0.01
AFP	3,265	7	0.002	0.02
ADE	3,239	7	0.002	0.02
AGR	3,315	11	0.003	0.03
AAW	97	1	0.01	1.0
AKR	331	11	0.03	3.0
ARQ	575	110	0.19	19
ALT	358	91	0.25	25
ACM	351	95	0.27	27
AXI	665	176	0.26	26

As shown in Table 2, 152,658 responses were expected for the entire student population. Of this number, 73906 responses were received, which constituted a response rate of 48.4%. This suggests that more than half of the responses were not received (51.6%), indicating a high rate of non-response. Concerning specific levels of study, it was observed that non-response was more prevalent among Level 200 students (25%), whereas non-response was less prevalent among Level 600 students. Based on these ratios, groups with large class sizes usually had low proportions of students completing or participating in the survey. This result is presented in graphical form in Figure 1.

As shown in Figure 2, the non-response rates for levels 900, 500, 300, and 200 were below 0.50. Among the postgraduate students (from level 700 to 900), level 700 students had a high response rate, followed by level 800 students, while non-response appeared to be more prevalent among level 900 students (doctoral students).

Specific courses were selected, and their response rates were explored. This strategy was used to clarify the results in Table 1.

As shown in Table 3, some courses had a response rate lower than 1%. For instance, a course such as ABO, which had an expected response of 3,246, recorded only six responses, resulting in a 0.01% response rate. The AFP and ADE courses recorded response rates of 0.02% each. While courses such as AGR recorded a response rate of 0.03%, others also recorded response rates between 25 and 27 (e.g., ALT, ACM, AXI).

### How unit non-response affects the dependability of students’ ratings

To address the second objective, we first computed the variance components for the identified sources of variation (G-study), followed by reliability estimates and measurement errors for the data through optimization.

#### Results from G-study analysis

The results of the G-study revealed that the largest source of rating variation in the evaluation of teaching was due to the residual [(rater: person) × item (***r**:**p***) ***i,e***] with a variance estimate of 1.167 and a variance percentage of 71.1 ( $\sigma^{2}$ =0.1.167, 71.1%). This suggests that the variability of students’ ratings of lecturers is influenced by the systematic interaction of raters (nested in classes) by items as well as other systematic and random factors that were not explored in this study. Raters nested in persons (***r*:*p***) had the second-largest variance contribution, with a variance of 0.250 and a corresponding percentage of 15.2. This result indicated that raters systematically differed in how they rated the same lecturer. Item ( $\sigma^{2}$ =0.011, 0.7%) had the least contribution to the variability in students’ ratings of lecturers, signifying that there was consistent use of the items among students from a single class (see Table 4).

**Table 4:** Sources of variability and their variance components for teaching appraisal.

Sources of variation	df	SS	MS	Variance	Variance percentage
person (*p*)	144	4,675	32.47	0.150	9.20
item (***i***)	9	300	33.28	0.011	0.70
person x item (***pi***)	1,296	3,019	2.33	0.063	3.80
rater: person (***r*:*p***)	2,556	9,374	1.67	0.250	15.2
(rater: person) x item (***r**:**p***)***i,e***	23,004	26,842	1.17	1.167	71.1

df, degrees of freedom; MS, mean square; SS, sum of squares.

#### D-study (optimisation)

An optimization analysis was conducted to model how non-responses affected the data provided by the students. The modeling was performed using non-response intervals of 1 (i.e., only one rater failed to respond; see the charts), 10 (i.e., 10 raters failed to respond), and 47 (i.e., 47 raters failed to respond).

**Figure 3:**
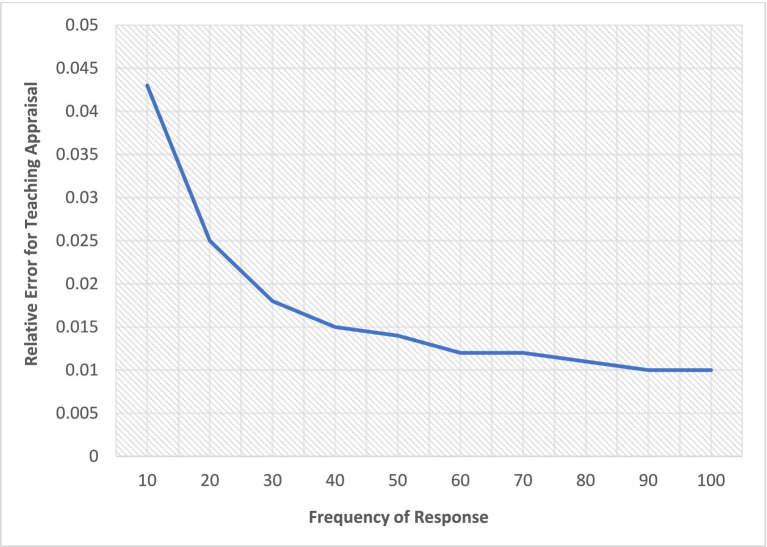
Trend analysis of frequency of non-response and relative error variance for appraisal of teaching. A line graph with relative error variance and frequency of responses at the x and y axis, respectively.

Table 5, together with Figures 2, 3, highlight how non-responses affect the online teaching evaluation data provided by students. It must be emphasized that 10 items were used for the evaluation of the teaching exercise.

**Table 5:** Influence of non-response on teaching evaluation data (*n*_i_ = 10).

No. of raters	Absolute error	Relative error	Phi	G
NR = 10				
100	0.011	0.010	0.932	0.938
90	0.011	0.010	0.929	0.936
80	0.012	0.011	0.926	0.933
70	0.013	0.012	0.923	0.929
60	0.013	0.012	0.918	0.924
50	0.015	0.014	0.911	0.917
40	0.017	0.015	0.901	0.907
30	0.020	0.018	0.885	0.891
20	0.026	0.025	0.854	0.860
10	0.044	0.043	0.774	0.778
NR = 47^a^				
236	0.009	0.008	0.951	0.951
189	0.009	0.008	0.948	0.948
142	0.010	0.009	0.938	0.944
95	0.011	0.010	0.931	0.937
48	0.015	0.014	0.909	0.915
1	0.374	0.373	0.287	0.288

NR, Non-response; *n*_i_, number of items.

a Average non-response of all cases.

The results revealed that a class of 100 students produced a generalizability index of 0.938 and an associated relative error of 0.010 (see Table 5). For example, if 30 students in the class failed to participate in the evaluation exercise, the reliability coefficient would reduce to 0.929, and the relative error would increase to 0.012.

**Figure 4:**
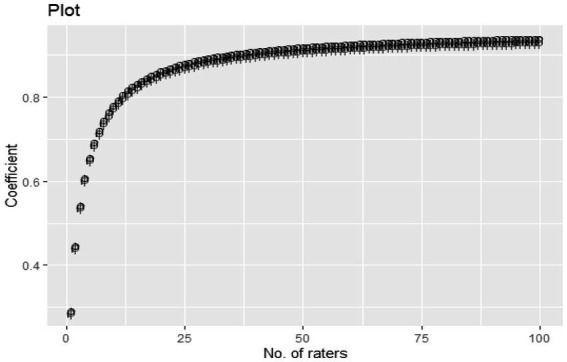
Trend analysis of number of raters and G/Phi coefficient for appraisal of teaching. A line chart showing the relationship between the number of raters and the associated reliability coefficient.

A similar trend of results was revealed (see Figures 3, 4), such that the more students failed to participate in the evaluation survey, the more the reliability of the data reduced and errors increased. Figure 3 shows that there is a negative relationship between the frequency of unit responses and errors associated with rating variability in the online teaching evaluation. In other words, increasing the response resulted in a decreasing relative error. When the number of responses for teaching evaluation decreases, the error increases relatively, resulting in low dependability of the online teaching evaluation data provided by students.

As shown in Figure 3, there appears to be a positive association between the number of raters and reliability coefficients (both *g* and *phi*) regarding teaching evaluation. It can be observed that as the number of raters decreased from 100 to 0 along the horizontal axis, both reliability indices also reduce from 0.80 to 0 along the vertical axis.

From the results, as more students drop out of the teaching evaluation exercise, the higher the ratings become inaccurate, and the final results are less dependable. The results also show that although a higher number of responses is preferable in terms of improving the quality of data obtained, having a minimum number of 50 students results in an acceptable reliability level (coefficient > 0.90).

## Discussion

Given the increasing global digitalization of students’ evaluation of teaching exercises, this study investigated the levels of non-response to online teaching appraisal surveys in higher education in Ghana. The study, through the GT approach, also modeled unit non-response rates with the dependability of the responses provided by students in this evaluation exercise. We discovered that unit non-response was common among students during the online teaching appraisal at the selected university. As such, the majority of the students did not participate in this exercise. This trend of result reflects the outcome of recent studies that have reported unit non-response as a common phenomenon in online surveys (Marcham et al., 2020; Čehovin et al., 2022; Falk and Thies, 2022; Plante et al., 2022). Nevertheless, the recorded high unit non-response rates in this context cannot be entirely attributed to the transition from a paper-and-pencil survey to an online survey. These low rates of participation could also be attributed to the non-availability of (or limited access to) internet and technological gadgets for use (e.g., internet-supported phones, laptops, etc.), and the high cost of internet data that prohibit internet usage in low- and middle-income countries, especially on issues such as teaching evaluation for which students might not see the direct benefits. This understanding is well anticipated within the Ghanaian context, as several empirical studies have documented the presence of numerous challenges with the use of technological means of teaching in higher education, which has been exacerbated by the COVID-19 pandemic (Agormedah et al., 2020; Abusamhadana et al., 2021; Adarkwah, 2021; Ankoma-Sey et al., 2022; Boateng and Tindi, 2022). Relatedly, technical issues concerning the online platform used for the administration of the survey instrument can also result in the challenges reported in earlier research. Concerns about the online administration platforms– such as difficulty accessing the website, unfriendliness of the platforms, and device compatibility– are critical for enhancing students’ experiences in responding to the survey. There is a need for continuous effort towards conducting research into exploring issues surrounding the friendliness and accessibility of the online teaching evaluation platforms. Institutional administrators are encouraged to rely on such information to leverage the rate of student engagement in online teaching evaluation surveys.

Remarkably, some studies have shown that students are motivated by the use of technology-driven platforms (such as Facebook, WhatsApp, YouTube, and Twitter) that offer direct gratification irrespective of the challenges associated with their usage (Owusu-Ansah et al., 2021; Quansah et al., 2022). This observation suggests that students are likely to find means of overcoming the challenges associated with technology use when they are aware of any associated direct and/or indirect benefits. Therefore, it is likely that student participation rates would increase when these students realize that the teaching evaluation research is relevant and that their responses can affect institutional decisions and practices. Although institutions do well to sensitize students to participate in evaluation exercises, when these benefits are not well communicated, demonstrated or felt by the students over a period, it will become difficult to achieve high participation as suggested by the selection bias model (Gronau, 1974; Heckman, 1974). This phenomenon leads to situations where students may show a lack of interest in participating in online assessment surveys because they consider the activity irrelevant. Even though the teaching evaluation survey can be masked as mandatory, it might not promote students’ interest and would only result in providing inaccurate responses. It is important that administrators of higher education institutions: (1) transparently communicate the purposes and benefits of the survey and strategize on how to ensure that students are aware of the relevance of their participation, (2) clearly communicate how the outcome of the teaching evaluation will be used; this approach may serve as an incentive for participation, and (3) show evidence of how previous teaching evaluation results have been used to improve teaching and learning within the institution.

A close analysis of how the online teaching evaluation is conducted in the selected university reveals the possibility of students experiencing fatigue in the process; this observation could plausibly explain the low participation rates (Spooren and Van Loon, 2012). As earlier stated, the said university administered a 25-item evaluation questionnaire, which appears somewhat appropriate and unlikely to lead to fatigue. However, students simultaneously responding to the questionnaire based on the number of courses they have registered could rather increase fatigue and boredom. By implication, students who have registered for 6 courses will be required to respond to the evaluation instrument 6 times at approximately the same time. This situation leads to student fatigue in answering the survey and consequently, lower response rates. Exploring ways to reduce fatigue in the administration of the evaluation questionnaire would help promote the participation of students in the evaluation survey.

It is instructive to add that the incidence of unit non-response in online teaching evaluation could be attributed to the sensitive nature of the items. For example, questions involving the ratings of teaching quality or teacher performance could prevent filling out the questionnaire for fear of negative repercussions. Concerns of this nature occur when there is the existence of a negative critical culture (e.g., such as fear of reprisals or feeling discouraged from sharing honest opinions) in the institution. This culture becomes worse when key ethical considerations such as anonymity and confidentiality are not prioritized and this may prevent students from providing candid responses. A culture of constructive feedback should be encouraged, and strengths valued in higher education institutions by enlightening all parties (i.e., lecturers, students and society) about the essence of the teaching evaluation to the growth of the institution.

Interestingly, varying levels of unit non-response were observed across the different registered courses. The differences in the participation rate, for example, could be attributed to the course characteristics (i.e., course content difficulty and students’ satisfaction with learning outcomes), the instructor characteristics (i.e., pedagogical strategy) and the context in which the course content is unpacked (i.e., negative critical culture, availability of resources/equipment during instruction) (Adams and Umbach, 2012). However, since the course names in this study were replaced with pseudonyms for confidentiality reasons, it is difficult understanding the nature of courses concerning the participation rates. It is important for course types and their associated rates of participation to be studied over a period of time in future studies to offer more insights into how these characteristics influence students’ participation in teaching evaluation.

This study further revealed that the frequency of unit non-response was negatively related to the accuracy of the measurement of teaching quality. That is, a high level of non-response resulted in a high rate of measurement error and a low level of validity of the responses provided by students. This result implied that the more students withdrew their participation in the online teaching evaluation, the blurrier the “picture” of the instructors created. It has been found that a unit of non-response results in a random error that affects the quality of data obtained (Dillman et al., 2002); thus, the least non-response should not be taken for granted, especially when the evaluation results for course/instructor are interpreted based on the responses from a normative group. This finding confirms reports from previous pieces of research, which also revealed differential responses from students who participate in the evaluation exercise and those who do not (Reisenwitz, 2016; Goos and Salomons, 2017). Other scholars also reiterated the discrepancies in the variances in the outcome of teaching evaluation surveys with high response levels and those with low participation rates (Bacon et al., 2016; Luo, 2020). Despite the use of distinct approaches in these previous studies, a common conclusion is communicated– the level of non-response influences the accuracy of the outcome of the teaching evaluation.

A key finding worthy of emphasis is that having approximately 50 or more students in a class who respond to the teaching evaluation survey would be likely to yield appreciable and more representative and accurate evaluation data. This notion suggests that for courses with class sizes far larger than 50 students, say 150, some level of non-response is permitted, yet the validity of the data is assured. The challenge, however, would be for courses with fewer students (less than 50 students, say 25), as in such classes, a 100% response rate could be obtained and yet responses would not be comprehensive and reliable enough to reflect the quality of teaching. This assertion has been confirmed in previous studies, stressing that small classes usually produce high-rating variances (Chang and Hocevar, 2000; Kane and Staiger, 2002). This finding calls for a more qualitative means of evaluation (e.g., interviews, open-ended questions) for small class sizes to supplement the use of close-ended items. Similar to the findings of this study, other studies have also recommended 20 student-raters as ideal in order to obtain a sufficient level of dependability (Li et al., 2018; Quansah, 2020). The results of these earlier studies contradict that of the present study, probably because of the differences in the number of facets used. There is, therefore, a need for future studies to continue the discourse on the number of students required to rate the quality of teaching.

The selection bias model provides greater insight into the findings of this research by emphasizing that bias can occur when teaching evaluation data for a course are provided by a non-random cross-section of students instead of the general student population enrolled in the course (Gronau, 1974; Heckman, 1974). Given this view, the finding that low rates of unit non-response are associated with low reliability of responses could be explained by the fact that the few students who participated in the exercise possessed some characteristics in terms of motivation, academic achievement, or personality, which influenced them to respond to the survey. Conversely, those who fail to respond to the evaluation form may also have similar unique traits that motivated their non-participation. Undoubtedly, these two groups of students may provide different responses and the evaluation outcome for any of the groups will not be representative of the student population of interest (Goos and Salomons, 2017; Luo, 2020). The selection bias model presents an additional perspective to the results that the low response rate observed in this study could be a reflection of the satisfaction the students derive from responding to the evaluation. For example, if students feel that the responses they provide are not utilized by the university administrators, as expected, the majority of such students are more likely to withdraw their participation.

### Practical implications

The findings of this study underscore the relationship among non-response, reliability, and measurement errors. Accordingly, higher levels of non-response resulted in lower reliability estimates and higher rates of measurement errors. This finding has implications for the accuracy of data obtained for decision-making during student evaluation of teaching in higher education, especially when there are low participation and response rates. Higher education administrators must embark on sensitization and awareness exercises for students on the need to actively participate in the appraisal of teaching to address the issue of non-response. This exercise should be performed, particularly in orientation sessions for fresh students newly admitted to the university (if not done). These forms of training should go beyond making the students aware of the existence of a directorate/unit in charge of the online teaching evaluation but rather enlighten the students on the benefits of being part of the evaluation. For continuing students, the directorate/unit can assign some staff to various classes to meet with the students for sensitization before the evaluation is carried out. In all these strategies, one thing should be paramount; that is, emphasis should be placed on educating students to religiously partake in the evaluation exercise and also expose them to the implications associated with non-participation.

Furthermore, higher education administrators should create opportunities for students to conveniently participate in evaluation exercises without any stress or fatigue. Most importantly, internet availability, accessibility, and internet gadget availability should be a priority for the management of higher education institutions. Perhaps, the management of higher education institutions can explore avenues that provide incentive or valuation strategies for participating in this evaluation exercise. It is worth emphasizing that university students and professors have a critical role to play in ensuring that minimal measurement errors are introduced in the teaching evaluation data. Students are expected to demonstrate heightened motivation towards participating in teaching evaluation surveys, and as well represent themselves well during the exercise by providing accurate responses regardless of the situation they find themselves. Professors have a role to play in terms of encouraging and sensitizing students to participate in the teaching evaluation survey through feasible strategies such as promoting a culture of constructive feedback.

### Strengths

This research adds to the existing literature on the role of unit non-response in evaluation surveys by providing insights into the extent of misrepresentations caused by this phenomenon. The unique feature of this study lies in the approach adopted and its ability to provide limits on the number of students required to offer a high level of validity and low measurement errors in terms of the responses provided. For example, the study found that 50 or more students in a course were likely to provide accurate and reliable responses in evaluating teaching quality.

The study findings provide relevant information that benefits administrators, students and lecturers/instructors in higher education institutions. While the findings offer insight into why students and instructors should contribute to improving participation and accuracy of teaching evaluation data, administrators are also enlightened on the need to adopt strategies that promote high rates of participation. A much broader social impact of this research makes it useful for researchers who conduct survey studies irrespective of their field of investigation. Given that non-response positively relates to measurement errors and is negatively associated with the reliability of responses, these researchers would become aware of the implications associated with any recorded low participation in their research. Thus, this understanding would guide them to adopt measures to increase participation and as well be guided on how results from their research are interpreted.

### Limitations and future directions

Despite the significance of this study, it has some limitations. First, the data used were only for a single semester (i.e., the second semester of the 2019/2020 academic year); thus, the results may not be sufficiently representative for generalization. Second, the data obtained did not include demographic characteristics (e.g., gender, age, course major, department/faculty, grade point average); therefore, some relevant information that could have helped to better understand the results was not available.

Future studies should apply the GT approach to longitudinal teaching evaluation data to better understand the issue of non-response from one semester to another. A more detailed approach to discussing the causes of non-response is warranted, and further research should conduct a follow-up by identifying students who did not respond to the evaluation and interviewing them to determine and clarify the reasons for their non-participation. Moreover, scholars are encouraged to study non-responses and how they relate to demographic characteristics.

## Conclusion

The research highlights that high prevalence rates of non-participation in online teaching evaluation surveys in higher education are associated with inaccurate descriptions and misrepresentation of the quality of teaching. This study reported a low level of participation in the online teaching evaluation; this raises several questions regarding the soundness of the interpretations and use of the evaluation results. This outcome has consequences for the use of data in terms of informing institutional policies, professional development training, and promotional decisions. Essentially, the lecturers and professors would also have a share of the effect of unit non-response by receiving unfair ratings that may not reflect their teaching practices and quality assessment. Lessons learnt from this research signal a shared responsibility by students, professors (instructors) and higher education institutions in ensuring that there is high participation and candid responses during online teaching evaluation surveys.

## Data availability statement

The raw data supporting the conclusions of this article will be made available by the authors, without undue reservation.

## Ethics statement

The studies involving humans were approved by Institutional Review Board (IRB), University of Cape Coast, Ghana. The studies were conducted in accordance with the local legislation and institutional requirements. Written informed consent for participation was not required from the participants or the participants' legal guardians/next of kin in accordance with the national legislation and institutional requirements.

## Author contributions

YD: investigation, methodology, validation, visualization, writing – original draft, and writing – review & editing. FQ: conceptualization, data curation, formal analysis, investigation, methodology, software, validation, visualization, writing – original draft, and writing – review & editing. All authors contributed to the article and approved the submitted version.

## Conflict of interest

The authors declare that the research was conducted in the absence of any commercial or financial relationships that could be construed as a potential conflict of interest.

## Publisher’s note

All claims expressed in this article are solely those of the authors and do not necessarily represent those of their affiliated organizations, or those of the publisher, the editors and the reviewers. Any product that may be evaluated in this article, or claim that may be made by its manufacturer, is not guaranteed or endorsed by the publisher.
