# Supplementary material for: Modeling unit non-response and validity of online teaching evaluation in higher education using generalizability theory approach
Source: Front Psychol. 2023 Sep 4;14:1202896. doi: 10.3389/fpsyg.2023.1202896 (PMC10507623; doi:10.3389/fpsyg.2023.1202896)
Supplement: Supplementary file 1 [file Table_1.DOCX]

# SAMPLE OF EVALUATION FORM

The University takes a survey every semester to appraise their lecturers and their courses. Every student taking a course in the University, is supposed to complete a survey for all the courses that he/she takes.

Note that the information you provide during this process will be kept strictly **confidential**. Your **name** and **index number** will be kept anonymous. By **honestly** and **candidly** completing this survey, you would be providing this University with valuable feedback for improvement on the quality of education.

Course Outline
Please tick only one of the options as applicable

1. The lecturer made a course outline available to students at the beginning of the course

 No Yes

Course Content
Please tick only one of the options as applicable

2. The course content was based on the outline provided

 Less than 70% 70 - 79% 80 - 89% 90% or more

3. The course content is likely to be covered at the end of the teaching period.

 Not likely Slightly likely Likely Very likely

4. The course content was detailed (i.e each week with its specific content, materials to be read, etc)

 Not detailed Slightly detailed Detailed Very detailed

Attendance
Please tick only one of the options as applicable

5. The lecturer met the class ...

 Less than 70% of the time 70 - 79% of the time 80 - 89% of the time 90% or more of the time

6. The lecturer was most often in class ...

 15 minutes or more late 10 -14 minutes late 5 - 9 minutes late On time

7. The lecturer most often left the class ...

 More than 15 minutes to time 10 -14 minutes to time 5 - 9 minutes to time At the end of the period

Mode of Delivery
Please tick only one of the options as applicable

8. The lecturer demonstrated knowledge of the subject matter

 Not very well Not well Well Very well

9. The lecturer's delivery was well organised and systematic

 Not well Not very well Well Very well

10. The lecturer effectively communicated what he/she was trying to teach

 Not very well Not well Well Very well

11. The lecturer used class time to fully promote teaching and learning

 Not very well Not well Well Very well

12. The lecturer encouraged students to read materials beyond the suggested readings and lecture notes

 Not very well Not well Well Very well

13. The lecturer encouraged students to analyse issues from different perpectives

 Not very well Not well Well Very well

14. The lecturer accepted other points of view

 Not very well Not well Well Very well

15. The lecturer made time during lecturers for questions and discussions

 Not very well Not well Well Very well

16. The lecturer's responses to questions were appropriate and informative

 Not very well Not well Well Very well

17. The lecturer was concerned with the academic progress of students

 Not very well Not well Well Very well

Assessment
Please provide a candid response

18. Indicate the mode(s) of continuous assessment used in the course

 Quizzes Assignments Presentation Term paper

19. The number of assessments given by the lecturer was

 0 1 2 or more

20. The lecturer graded assessments

 No Yes

21. The graded assessments were returned in a good time

 No Yes

22. The graded assessments were subsequently discussed

 No Yes

Comments
**Please indicate briefly**

23. Lecturer's strength(s)

(320 characters remaining)

24. Lecturer's weakness(es)

(320 characters remaining

25. Any other suggestions
